# Radiation Sensitivity of Adipose-Derived Stem Cells Isolated from Breast Tissue

**DOI:** 10.3390/ijms19071988

**Published:** 2018-07-07

**Authors:** Annemarie Baaße, Friederike Machoy, Dajana Juerß, Jana Baake, Felix Stang, Toralf Reimer, Björn Dirk Krapohl, Guido Hildebrandt

**Affiliations:** 1 Department of Radiotherapy and Radiation Oncology, University Medical Centre Rostock, Suedring 75, 18059 Rostock, Germany; friederike.machoy@uni-rostock.de (F.M.); dajana.juerss2@uni-rostock.de (D.J.); jana.baake@uni-rostock.de (J.B.); guido.hildebrandt@uni-rostock.de (G.H.); 2 Clinic for Plastic, Hand and Reconstructive Surgery, University Hospital Schleswig-Holstein, Campus Luebeck. Ratzeburger Allee 160, 23538 Luebeck, Germany; felix.stang@uksh.de; 3 Department of Obstetrics and Gynecology, University of Rostock, Women’s Hospital, Suedring 81, 18059 Rostock, Germany; toralf.reimer@med.uni-rostock.de; 4 Berliner Centrum für Musikermedizin, Universitätsmedizin Berlin, Charitéplatz 1, 10117 Berlin, Germany; bjoern-dirk-krapohl@charite.de

**Keywords:** adipose-derived stem cells, radiation therapy, ionizing radiation, regenerative medicine

## Abstract

Within their niche, adipose-derived stem cells (ADSCs) are essential for homeostasis as well as for regeneration. Therefore, the interest of physicians is to use ADSCs as a tool for radiation oncology and regenerative medicine. To investigate related risks, this study analyses the radiation response of adult stem cells isolated from the adipose tissue of the female breast. To avoid donor-specific effects, ADSCs isolated from breast reduction mammoplasties of 10 donors were pooled and used for the radiobiological analysis. The clonogenic survival fraction assay was used to classify the radiation sensitivity in comparison to a more radiation-sensitive (ZR-75-1), moderately sensitive (MCF-7), and resistant (MCF10A) cell lines. Afterwards, cytotoxicity and genotoxicity of irradiation on ADSCs were investigated. On the basis of clonogenic cell survival rates of ADSCs after irradiation, we assign ADSCs an intermediate radiation sensitivity. Furthermore, a high repair capacity of double-strand breaks is related to an altered cell cycle arrest and increased expression of cyclin-dependent kinase (CDK) inhibitor p21. ADSCs isolated from breast tissue exhibit intermediate radiation sensitivity, caused by functional repair mechanisms. Therefore, we propose ADSCs to be a promising tool in radiation oncology.

## 1. Introduction

The stromal vascular fraction of adipose tissue (AT) represents a source of multipotent stem cells called adipose-derived stem cells (ADSCs) [1,2]. Since their first isolation by Rodbell in 1964 [3], advantages of ADSCs in clinical applications have become more present in the past years. First, in contrast to embryonic stem cells (ESCs) and induced pluripotent stem cells (iPSCs), adult stem cells are naturally immune-compatible and there are no ethical issues related to their use. Second, AT as a source of adult stem cells is easily accessible compared to the more invasive and severe pain-associated bone marrow harvesting. In addition, AT from liposuctions or breast reductions is often discarded as medical waste, so no additional surgery is required. Simultaneously, ADSCs and bone marrow-derived stem cells (BMSCs) bear similar phenotypes, including their capacity to self-renew, specific panels of surface proteins, and similar differentiation potentials [2,4,5]. Consequently, ADSCs seem to represent a more promising tool for regenerative therapies than BMSCs [6,7,8]. From this reasoning, the characterization and usage of ADSCs for clinical applications have recently been the focus of research.

Some applications of AT have been routinely used for decades, such as mammary lipografting to patients for breast reconstructive and cosmetic purposes [9]. However, to receive satisfying results, several repetitions of the lipografting procedure have to be performed [9], because only about 30–40% of the transplanted cells survive this procedure [10], due to the initially missing vasculature of the transplant, which leads to the hypoxic situation and thus to the increased death rate of the cells within the lipograft. One solution is proposed by the working group around Adachi, who achieved an increase of MMP-2 (matrix metalloprotenase-2) and VEGF (vascular endothelial growth factor) by low-dose irradiation (IR) of ADSCs; these signaling agents are known to increase angiogenesis in target cells. This way, the IR procedure of ADSCs might improve the cell survival of the autologous lipograft [11].

What has not yet been considered, however, is the risk of undesired side effects after radiation treatment of ADSCs. Therefore, this study investigates the cytotoxic effects of radiation on pooled ADSCs (pADSCs) as well as their repair capacity of DNA damage. In the course of radiation therapy, high energy is transferred either directly onto DNA molecules or indirectly by producing free radicals, which in turn affect the DNA strands. The result may also be lesions on the DNA bases or the sugar backbone, but the main cause of cell death is double-strand breaks (DSBs) [12]. Therefore, repair mechanisms of DSBs are crucial for cells to overcome radiation-induced cell lesions. Depending on the extent of cell damage, either apoptosis or cell cycle arrest is initiated, whereby the latter is being used to initiate repair mechanisms. In both directions, p53 was identified as the main regulator depending on activated target genes. While p53 regulates apoptosis through transcriptional activation of the bcl2 family members (bax, noxa, and puma) [13], the cell cycle arrest and the subsequent entry into repair mechanisms is targeted through the cyclin-dependent kinase (CDK) inhibitor p21 [14]. Those repair pathways have already been confirmed for mouse and human BMSCs [15,16] as well as for ADSCs isolated from the mouse [17]. Whether, as expected, similar radiation-induced mechanisms are functional in human breast-derived ADSCs, is to be investigated within this study.

Being aware of the extent of possible damages to ADSCs after beam exposure may also be important for planning radiation treatment of patients, because they are crucial for the healing of many tissue damages. Within their niche, ADSCs are essential for homeostasis of adipose tissue through adipogenesis and angiogenesis. Activated by external signals, ADSCs are able to secrete factors to induce adipose remodeling and neovascularization in damaged tissue [18,19,20]. Therefore, the management of radiation therapies which do not cause extensive damage in ADSCs could reduce the incidence of undesirable side effects. 

Thus, the purpose of this study is to classify the radiation sensitivity of ADSCs isolated from the breast and to clarify underlying mechanisms.

## 2. Results

### 2.1. Immunophenotype of pADSCs Does Not Change after IR with a Maximal Dose of 8 Gy

For this study, ADSCs were obtained from human reduction mammoplasties from healthy female donors. As shown in a previous study, these cells are characterized by a spindle-shaped and fibroblast-like morphology as well as their ability to adhere on plastics. Furthermore, the cells exhibit a multilineage capacity and a specific panel of expressed and lacking surface antigens (CD29^+^, CD90^+^, CD31^−^, CD34^−^, CD45^−^, CD106^−^) [21]. 

**Figure A1:**
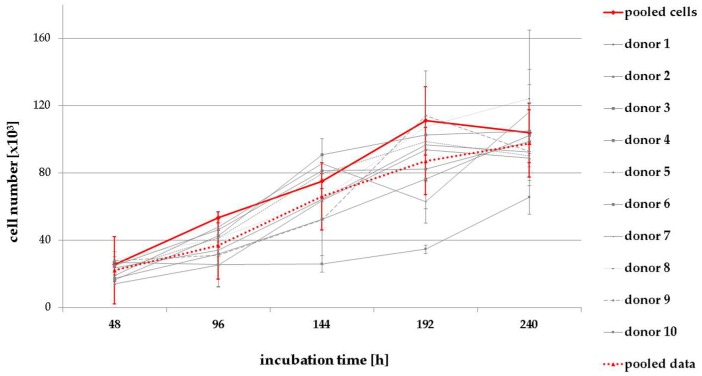
Donor-specific differences in the growth rate of ADSCs, isolated from ten different donors: comparison of pooled cells and pooled data. Results are illustrated as mean ± standard deviation (SD, *n* = 2).

**Figure A2:**
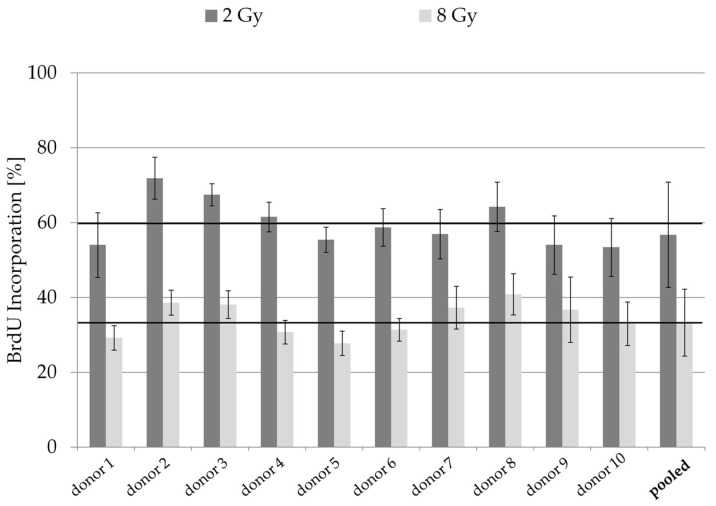
Donor-specific differences in the proliferation rate of irradiated ADSCs: comparison of pooled cells and pooled data of ten different donors. Cell proliferation was measured using a BrdU ELISA colorimetric assay. Black lines illustrate the mean value of all individual donors. Results are illustrated as mean ± standard deviation (SD, *n* = 3).

**Figure A3:**
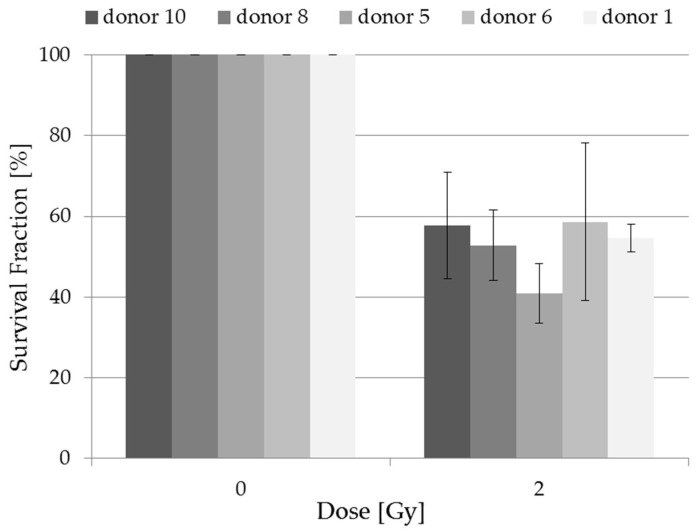
Donor-specific differences in the survival fraction of irradiated adipose-derived stem cells. The cells were seeded 24 h before the IR procedure and stained 20 days later by crystal violet to visualize formed colonies. The cell survival fractions (SF) of the different experimental approaches were normalized to those of unirradiated cells; *n* = 3 presented as mean ± standard deviation (one sample *t*-test).

**Figure A4:**
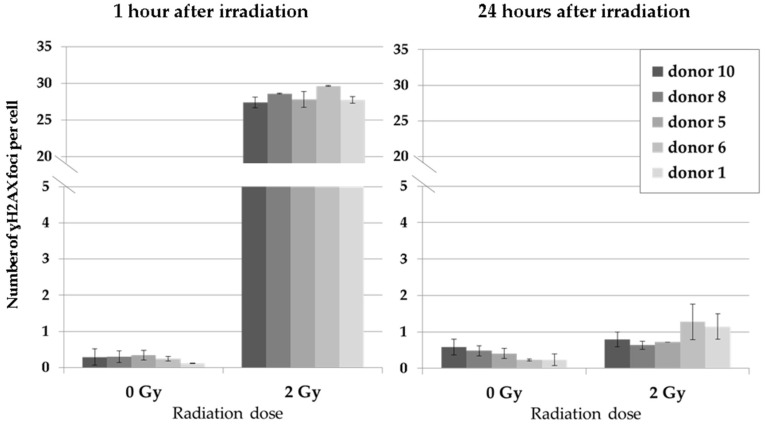
dsDNA-damaging effects of irradiation (IR) on ADSCs of individual donors and their repair capacity within 24 h. Results are illustrated as mean ± standard deviation (SD, *n* = 2).

In this study, ADSCs isolated from 10 different donors were pooled (pADSCs) to avoid donor-specific effects. Their growth kinetics, proliferation rate, survival fraction, and repair capacity of DNA double-strand breaks after the irradiation procedure were comparable to the average of the single analysis of each donor (Appendix, Table A2, Figure A1, Figure A2, Figure A3 and Figure A4). However, it should be noted that slight donor-specific differences exist in the growth rate of ADSCs. Therefore, pADSCs of a relatively high number of donors were chosen for pooling, and experiments were performed only a few doubling times after cell pooling.

**Figure 1:**
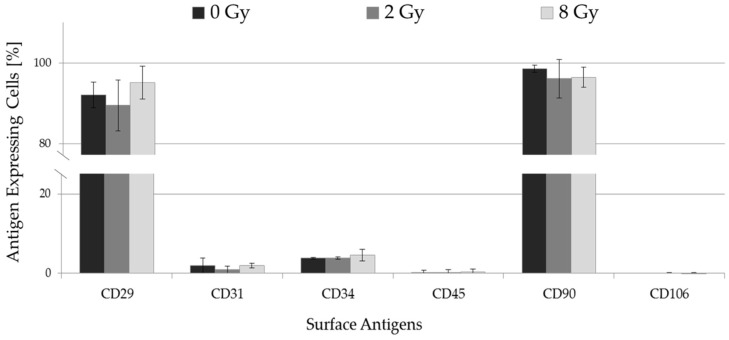
Surface antigen expressions (CD29, CD31, CD34, CD45, CD90, and CD106) of pADSCs 72 h after being irradiated with 2 or 8 Gy. The expression values of isotype-matched control immunoglobulin-labeled cells were deducted from determined expression levels of each surface antigen, and unstained cells were also carried as controls. Data are presented as mean ± standard deviation, *n* = 3.

As a basis for this study, the immunophenotype of irradiated pADSCs was characterized by expressed and lacking surface antigens. Seventy-two hours after IR with a dose of 2 or 8 Gy, pADSCs constantly expressed CD29 (90.0% and 95.7%) and CD90 (96.2% and 96.5%) in nearly every way that CD31 (1.3% and 2.4%), CD34 (4.3% and 6.6%), CD45 (0.6% and 0.7%), and CD106 (0.3% and 0.4%) were not (Figure 1). No significant differences relative to unirradiated cells were detectable. In summary, with a maximum IR dose of 8 Gy, the immunophenotype of pADSCs did not change after IR.

### 2.2. pADSCs Exhibit Intermediate Radiation Sensitivity

**Figure 2:**
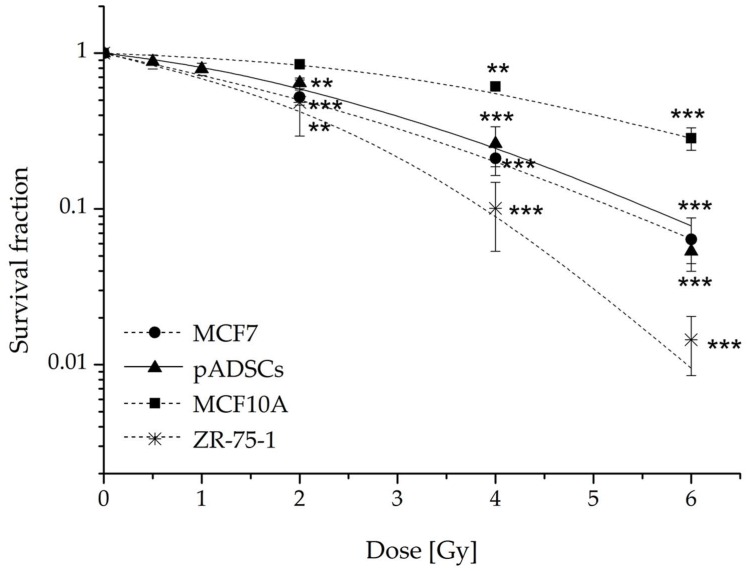
Colony-forming efficiency assay of pooled adipose-derived stem cells (pADSCs) in comparison to MCF-7, MCF10A, and ZR-75-1 cells. ADSCs of 10 donors were pooled and, like ZR-75-1, MCF-7, and MCF10A cells, seeded 24 h before the IR procedure, where 0 Gy was defined as the control. The cells were stained by crystal violet to visualize formed colonies. The cell survival fractions (SF) of the different experimental approaches were normalized to those of unirradiated cells; *n* = 5 (MCF-7 cells and ZR-75-1 cells), *n* = 4 (pADSCs), or *n* = 3 (MCF10A cells) presented as mean ± standard deviation. Asterisks illustrate significance: ** *p* < 0.01; *** *p* < 0.002 (one sample *t*-test).

In order to classify the radiation sensitivity of ADSCs, the radiation-sensitive breast cancer cell line ZR-75-1, the more moderately sensitive breast cancer cell line MCF-7 [22], and the rather radiation-resistant cell line MCF10A [22] were tested for their clonogenic survival fraction (SF) parallel to the analysis of pADSCs. The observed SF of the reference cell lines (Figure 2) are consistent with published data [22,23]. Additionally, we tested the nontumorigenic epithelial cell line MCF10A in order to compare the radiation sensitivity of pADSCs with a normal adjacent cell type. In general, the number of ZR-75-1, MCF-7, MCF10A, and pADSCs colonies decreased with increasing IR dose, whereby the survival curve of pADSCs runs between that of MCF10A and MCF-7 cells. An already low-dose IR of 0.5 Gy leads to a reduction of pADSC SF to 88 ± 9%. After IR with a dose range of 4 to 8 Gy, pADSCs and MCF-7 cells show comparable SFs, whereas pADSCs are less affected than MCF-7 cells after low-dose irradiation of 2 Gy (Appendix, Table A1). It should be emphasized that such an irradiation dose of 2 Gy is of particular clinical importance, since it is used for conventionally fractionated whole-breast irradiation of early stage breast cancer patients. Compared to MCF-7 cells and pADSCs, the nontumorigenic epithelial cell line MCF10A is rather radiation-resistant and the tumorigenic cell line ZR-75-1 is rather radiation-sensitive. Altogether, pADSCs exhibit intermediate radiation sensitivity. 

### 2.3. Irradiation Alters Cell Cycle Progression of pADSCs, Whereby p21 Was Identified as a Possible Mediator

#### 2.3.1. Irradiation Inhibits the Proliferation of pADSCs Even at Moderate Doses

**Figure 3:**
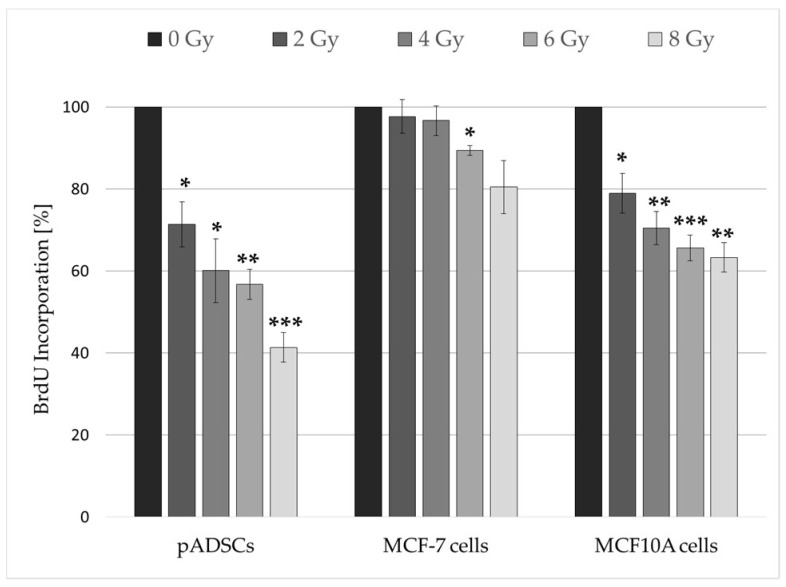
Proliferation rate of MCF-7 cells, pADSCs, and MCF10A cells within 48 h after the irradiation procedure. Cell proliferation was measured using a BrdU ELISA colorimetric assay. Results are illustrated as mean ± standard deviation (SD, *n* = 3). Asterisks illustrate significance: * *p* < 0.02; ** *p* < 0.01; *** *p* < 0.002 (one sample *t*-test).

As early as an IR dose of 2 Gy, the proliferation rates of pADSCs and MCF10A cells are significantly reduced, whereas an effect in MCF-7 cells could not be detected until a dose of 6 Gy (Figure 3). In contrast, the more radiation-resistant MCF10A cells react similarly to pADSCs, with an increasing inhibition of the proliferation rate with increasing IR dose, although the extent of this effect varies. The lowest inhibition was recorded for the tumorigenic MCF-7 cells, which were identified as a more radiation-sensitive cell line in the previous colony-forming units assay (see Section 2.2). Declining proliferation rates can be caused by two factors: (1) Irradiation causes apoptosis, so that the cell can no longer reproduce; (2) the repair of radiation-induced DNA damage is associated with the short-term stop of cell cycle progression and the transition to the cell cycle arrest. Therefore, relatively constant proliferation rates and low survival fractions can be explained by late or inefficient DNA repair mechanisms.

Altogether, pADSCs showed the strongest decrease in proliferation rate after IR. The reason for this could be an early initiation of apoptosis or the cell cycle arrest, the latter being associated with the introduction of DNA repair mechanisms. 

#### 2.3.2. Irradiation Exposure Induces the ADSCs to Move from the S-Phase into Cell Cycle Arrest

**Figure 4:**
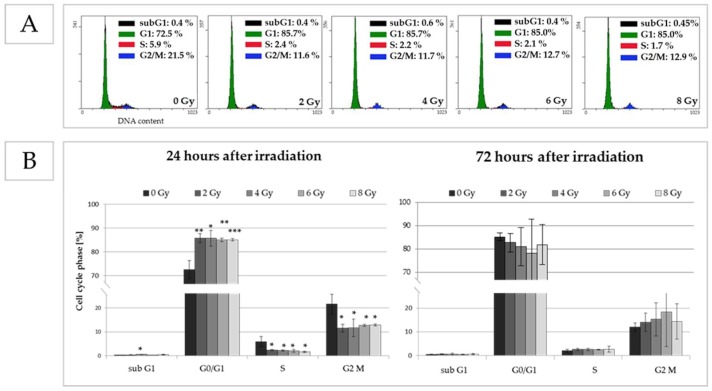
Effect of irradiation on cell cycle progression after an incubation time of 24 and 72 h. Distribution of cells in cell cycle phases (G0/G1, S and G2/M) and cells with degraded cell DNA (subG1) was determined by flow cytometry using propidium iodide (PI) to measure the DNA content of each cell. (**A**) Exemplary histograms of cell cycle assay by flow cytometry of unirradiated (0 Gy) and irradiated pADSCs (0.25, 1, 2, 4, and 6 Gy), 24 h after the irradiation procedure; on the right corner of each image, results are illustrated as mean ± standard deviation (SD, *n* = 3); (**B**) Graphical illustration of cell cycle distribution of unirradiated and irradiated cells; asterisks illustrate significant differences to unirradiated cells (control): * *p* < 0.05; ** *p* < 0.01; *** *p* < 0.001 (students *t*-test and two-way ANOVA with Bonferroni post-hoc test).

In response to IR, short-term effects on the cell cycle progression were detectable in pADSCs (Figure 4B, left). While the percentages of unirradiated cells in G1, S, and M phases were 73%, 6%, and 22%, respectively, a significant decrease to 2% of cells in the S-phase was observed 24 h after IR with application of 2 to 8 Gy, whereas the percentage of cells in G0/G1 phases numerically increased (2 Gy: 86%, 4 Gy: 86%, 6 Gy: 85%, 8 Gy: 85%). This indicates that the G0/G1 cell cycle checkpoint is functional in pADSCs that enter G1 cell cycle arrest. At a later time point of 72 h after IR, differences between irradiated and unirradiated cells are no longer detectable as early DNA repair mechanisms might be completed (Figure 4B, right). 

#### 2.3.3. The Protein p21 Could Be One Mediator of Observed Cell Cycle Progressions

**Figure 5:**
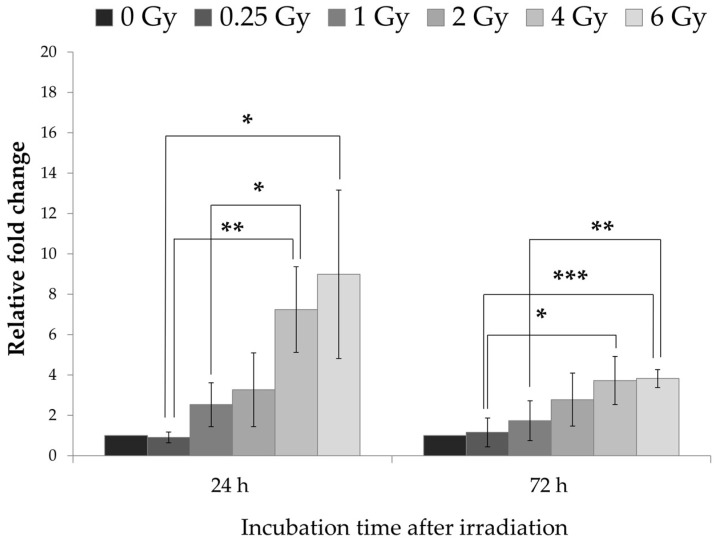
Influence of irradiation on gene expression of p21 in ADSC cells at different time points. Using the ΔΔC*_t_* method, data from three independent experiments were presented as mean of the relative expression values ± standard deviation. Asterisks illustrate significance: * *p* < 0.05; ** *p* < 0.01; *** *p* < 0.001.

The gene of the CDK inhibitor p21 is known to be a critical mediator of p53-dependent G1 arrest in tumor cells and the subsequent entry into repair mechanisms [24,25]. Here, we detected a raise of the p21 gene expression in pADSCs after IR, whereby a direct linearity was observable with increasing radiation dose (Figure 5). These effects are detectable both 24 h and 72 h after IR with a time-dependent attenuation of p21 expression in pADSCs after 4 and 6 Gy IR (two-way ANOVA, *p* < 0.001). Consequently, p21 could be one mediator of observed IR-dependent cell cycle progressions in pADSCs, as already demonstrated in BMSCs [26]. 

### 2.4. pADSCs Possess a High Repair Capacity of DNA Double-Strand Breaks

As observed here, pADSCs exhibit intermediate radiation sensitivity. Subsequent analysis of proliferation rate, cell cycle progression, and p21 expression suggest that relatively early repair mechanisms are introduced into these cells. To further investigate this hypothesis, IR-induced DNA damage was verified in the frequency of DSBs, both shortly after irradiation, to detect DNA damage, and after an incubation time of 24 h after IR, to analyze their repair. 

**Figure 6:**
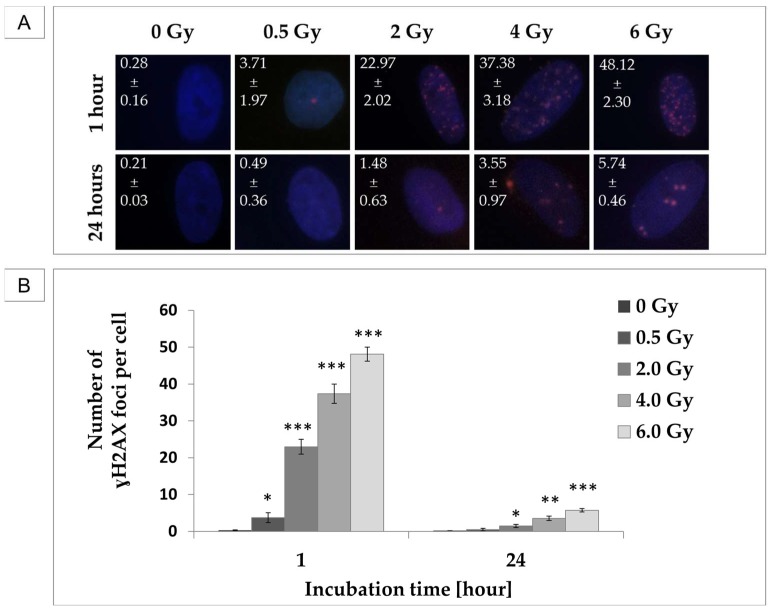
dsDNA-damaging effects of irradiation (IR) on pADSCs and their repair capacity within 24 h. Phosphorylated H2AX (γH2AX) was used as a marker for DNA double-strand breaks (DSBs). To determine γH2AX foci, cells were fixed 1 h or 24 h after IR and incubated with anti-γH2AX antibody and IgG1 (red); DNA counterstaining with 4,6-diamidino-2-phenylindole (DAPI) (blue). (**A**) Exemplary images of immunocytochemistry staining at different time points after IR. On the left corner of each image, results are illustrated as mean ± standard deviation (SD, *n* = 3); (**B**) Graphical illustration of mean number of γH2AX foci per cell; asterisks illustrate significant differences to unirradiated cells (control): * *p* < 0.05; ** *p* < 0.01; *** *p* < 0.001.

IR induced DSBs in pADSCs, whereby their occurrence increased in a linear way with increasing radiation dose (Figure 6). After an incubation time of 24 h, the level of DSBs in pADSCs decreased extremely, so that differences among unirradiated and 0.5 Gy-irradiated cells were not detectable. Even the 6 Gy IR-induced γH2AX foci decreased in number from 48 to 6 per cell nucleus after 24 h incubation. These findings implicate that the repair mechanisms of IR-induced DNA damage are functional in pADSCs within a dose range of 0.5 to 6 Gy.

## 3. Discussion

In the present study, we demonstrate that ADSCs isolated from the breast adipose tissue exhibit intermediate radiation sensitivity, as a result of early cell cycle arrest associated with increased p21 expression and fast DNA damage repair. 

These results correspond to the hypothesis that adult stem cells are more resistant to IR than embryonic stem cells. Nevertheless, tissue-specific differences exist, probably caused by disparities in the expression of pro-apoptotic or anti-apoptotic proteins as well as cell cycle duration and p53 dynamics [27]. Whereas stem cells from the bone marrow are well characterized for their radiation sensitivity [16], studies on ADSCs are lacking or are carried out with mice as the host [17]. In addition, there are characteristic differences between adult stem cells isolated from adipose tissues of different origins, including growth kinetics [28,29]—a factor that may be responsible for tissue-specific radiation sensitivities [27]. From this reasoning, extensive analyses of underlying IR-induced mechanisms in ADSCs from different origins have to be performed in the future to engineer reliable cellular therapy options in radiation oncology. 

To our knowledge, this is the first work that investigates the radiation response of ADSCs isolated from the breast and thus provides the first insights into unwanted side effects of radiation therapy that could result from damaged stem cells. Minimizing stem cell damage should be the aim of modern radiotherapy in order to reduce undesirable side effects. Here, we detected a full repair of DSBs only 24 h after an irradiation dose of 0.5 Gy. Due to higher IR doses, a small number of residual DSBs were observed. Whether repetitions of this IR procedure in the course of fractionated radiation therapy could be critical for the appropriate tissue is questionable. However, in BMSCs, fractionated doses of IR seem to be rather protective [30]. 

Altogether, further investigations are needed to determine the long-term effects of IR on ADSCs and especially the repeated IR procedure in the course of fractionated radiation therapy. Nevertheless, this study highlights the intermediate resistance of ADSCs isolated from the breast and their functional repair mechanism for IR-dependent damage. 

In conclusion, ADSCs isolated from the breast seem to be capable for cellular therapy options in radiation oncology and regenerative medicine before radiation therapy. 

## 4. Materials and Methods

### 4.1. Cell Culture

#### 4.1.1. Isolation of ADSCs

The isolation of ADSCs was performed with human reduction mammoplasties from healthy female donors. This work was approved by the ethics committee at the University of Rostock, Germany (registration-number: A201008). The protocol for the isolation of fat tissue has been developed and optimized from previously described work [23,31]. Tissue samples were mechanically minced and adipose tissue was washed three times with phosphate-buffered saline (PBS, PAN-Biotech GmbH, Aidenbach, Germany) and centrifuged (250× *g*). After each centrifugation, the infranatant and resultant pellet were removed. Samples were digested with 0.1% collagenase type I (100 U/mL, Gibco Life Technologies, Darmstadt, Germany) and supplemented with penicillin/streptomycin (P/S, 1%; 100 × penicillin 10,000 U/mL, streptomycin 10,000 µg/mL, Sigma-Aldrich, Steinheim, Germany) on a shaker at a low setting for 18 h at 37 °C. After complete dissociation, the tissue samples were washed with an equal volume of Dulbecco’s modified Eagle medium and Kaighn’s modification of Ham’s F12 (DMEM-F12 media, Life Technologies) and filtered through 100 µm strainers (Greiner Bio-One, Frickenhausen, Germany). This was followed by a centrifugation step (190× *g*, 10 min, 37 °C) to obtain the ADSC fraction. The supernatant was discarded and the pellet was resuspended in ADSC culture medium containing DMEM-F12 supplemented with 10% fetal bovine serum Superior (FBS, Biochrom AG, Berlin, Germany) and 1% P/S. Cells were cultured at 37 °C in the presence of 5% CO_2_ for 48 h in order to enable them to adhere. Afterwards, the non-adherent fraction was removed and the remaining cells were washed twice with PBS before seeding. The medium was replaced once every three days. When cells reached between 80% confluence, the medium was discarded, and cells were washed twice with PBS and trypsinized with 0.25% trypsin/EDTA (PAA Laboratories, Cölbe, Germany). The cell count was determined using a Coulter Z2 automated cell counter (Beckmann Coulter GmbH, Krefeld, Germany). The cells (passage 1) were cryopreserved in ADSC culture medium containing 10% dimethyl sulfoxide (DMSO; Merck, Darmstadt, Germany) and 20% FBS. Experiments were performed in passages three to five. 

To avoid donor depending effects of primary human cells, ADSCs of 10 different patients were cultured and pooled. Therefor the abbreviation pADSCs (pooled ADSCs) is used in the following.

#### 4.1.2. Cell Lines

For comparative purposes, two human breast cancer cell lines (MCF-7 and ZR-75-1) and the non-tumorigenic epithelial breast cell line (MCF10A) were tested parallel to the radiation biologic analyzes of pADSCs. The MCF-7 cells (HTB-22™) as well as the MCF10A cells (CRL-10317™) were purchased from the ATCC and the ZR-75-1 cells from ECACC. MCF-7 cells were cultured in DMEM (Lonza BioWhittaker, Verviers, Belgium) containing 1% P/S and 10% FBS at 37 °C with 5% CO_2_. MCF10A cells were provided by Prof. Kevin Prise, Queen’s University Belfast, Ireland, and cultivated using DMEM/F12 supplemented with 0.01% cholera toxin, 0.1% insulin, 0.05% hydrocortisone (all Sigma-Aldrich), and 1% P/S, 0.02% epidermal growth factor (EGF; Gibco/Life Technologies), and 5% horse serum (Fisher Scientific, Schwerte, Germany) under 5% CO_2_ and at 37 °C. ZR-75-1 cells were cultured in RPMI 1640 (PAN Biotech GmbH, Aidenbach, Germany) containing 10% FBS, 1% P/S, and 0.2% sodium pyruvate (Gibco Life Technologies, Darmstadt, Germany). 

#### 4.1.3. Cell Maintenance 

When cells reached between 80% confluence, the medium was discarded and cells were washed twice with PBS. Afterwards, ADSCs and ZR-75-1 cells were detached by using 0.25% trypsin/EDTA; MCF-7 and MCF10A cells by using 0.05% trypsin/EDTA. 

### 4.2. Irradiation 

The cells were irradiated 24 h after seeding using the Linac Siemens Oncor Expression (Healthcare Sector Siemens AG, Erlangen, Germany) at a dose rate of 3.75 Gy/min. Used IR doses were 0.25, 0.5, 2, 4, 6, and 8 Gy, where 0 Gy was utilized as the control.

### 4.3. Expression Panel of Characteristical Surface Proteins

For analysis of mesenchymal surface markers, ADSCs in passages three to five were trypsinized, washed with staining buffer (BD Pharmingen, BD Biosciences, Heidelberg, Germany), and stained with the following antibodies: CD29-PE, CD34-PE, CD90-FITC (all BD Biosciences), CD31-PE, CD45-PE, and CD106-PE (all Biolegend, London, UK) on ice and in the dark for 20 min. Fluorochrome-conjugated isotype control antibodies (BD Biosciences) were used to determine the level of nonspecific binding. Samples were washed (300× *g*, 4 °C, 5 min) and resuspended with staining buffer. The cells were analyzed by flow cytometry directly after incubation with 7-aminoactinomycin (7-AAD, Biolegend) for 10 min on ice and protected by light (Cytomics FC 500, Beckmann Coulter, Krefeld, Germany). Positive and negative events were calculated using the CXP™ software (Beckman Coulter) and gated for living cells (negative for 7-AAD).

### 4.4. Cytotoxic Effects of Radiation 

#### 4.4.1. Cell Proliferation Assay

Changes in the cell proliferation rate after IR were assessed by intracellular bromodeoxyuridine (BrdU) incorporation (Cell Proliferation ELISA, Roche Applied Science, Mannheim, Germany). For this purpose, 2000 pADSCs as well as 1500 MCF-7 cells and 2500 MCF10A cells were seeded in quintuplicate into 96-well plates (TPP Techno Plastic Products AG, Trasadingen, Switzerland). Those cell type-specific seeding densities were determined previously in order to avoid influencing the proliferative behaviour of cells by contact inhibition.

Radiation doses of 2, 4, 6, and 8 Gy were applied. After an incubation time of 48 h, which corresponds approximately to the determined doubling time of pADSCs, the colorimetric cell proliferation ELISA was performed according to the manufacturer’s instructions. In order to calculate the relative BrdU incorporation, the measurements of unirradiated cells were defined as 100% BrdU incorporation.

#### 4.4.2. Colony-Forming Units Assay 

To determine the long-term effect of IR on the cell survival, the colony-forming units assay was performed. Therefore, pADSCs (1000 cells), MCF-7 (500 cells), MCF10A (1000 cells), and ZR-75-1 cells cells (8000 cells) were seeded in triplicates in T_25_ flasks or 6-well plates (Greiner Bio One) and treated with the radiation doses ranging from 0 Gy to 6 Gy. A full medium exchange with culture medium was performed every three (ADSCs) or seven days (MCF-7, MCF10A, ZR-75-1). On day 7 (MCF10A), day 21 (ZR-75-1 cells), day 15 (MCF-7 cells), or on day 20 (pADSCs), the cells were stained by 1% crystal violet (Serva Electrophoresis GmbH, Heidelberg, Germany) to visualize formed colonies. Colonies consisting of at least 50 cells were counted by microscopy. The calculated plating efficiency (PE) and survival fractions (SF) were evaluated using the data analysis and graphics software Origin 8.6. 

### 4.5. Effects of Irradiation on Cell Cycle

#### 4.5.1. Flow Cytometry 

In order to analyze changes in cell cycle progression after IR, the DNA content in the cells was determined by flow cytometry. Twenty-four hours after IR (0, 2, 4, 6, and 8 Gy), pADSCs were harvested and fixed in 70% ice-cold ethanol at −20 °C overnight. Afterwards, the cells were incubated in 1 mL RNase A (100 μg/mL) at 37 °C for 15 min and stained with 50 µg/mL propidium iodide (Sigma Aldrich Chemie GmbH, Munich, Germany) at 4 °C overnight. Using Cytomic FC 500 flow cytometer and CXP analysis software, the DNA content was determined and analyzed. 

#### 4.5.2. Real-Time Quantitative Reverse Transcription-PCR

400,000 of pADSCs were seeded in T_75_-flasks and irradiated 24 h later. Total RNA was isolated 24, 48, and 72 h after IR using a NucleoSpin^®^ RNA kit (Macherey-Nagel, Düren, Germany). For cDNA synthesis, the RevertAid First Strand cDNA Synthesis Kit (Thermo Scientific, Schwerte, Germany) was used. The following Taq Man assays (Applied Biosystems^®^, Darmstadt, Germany) were used for the gene expression analysis: tumour protein p21 (TP21; Hs01040810_m1) as gene of interest and TATA-Box-Binding Protein (TBP; Hs00427620_m1) as internal control. Real-time quantitative reverse transcription PCR was performed using a PCR cycler AB7300 (Applied Biosystems^®^, Life Technologies, Darmstadt, Germany) under specific cycling conditions (1. 50 °C for 2 min, 1 cycle; 2. 95 °C for 10 min, 1 cycle; 3. 95 °C for 15 s to 60 °C for 1 min, 40 cycles). Every sample was tested in triplicate for three independent experiments. A normalization of the relative quantitative values of mRNA for tumor protein p21 (TP21) was performed to the endogenous control TBP via the ΔΔC*_t_* method. 

### 4.6. DNA Damaging Effects: Measurement of DNA Double-Strand Breaks (γH2AX Assay)

Twenty-four hours before IR, 35,000 pADSCs were seeded in duplicate in chamber slides (LabTek^®^, Nunc, Roskilde, Denmark). After fixation with 2% formaldehyde and permeabilisation with 0.25% Triton X-100 (both Sigma Aldrich Chemie GmbH), the cells were consecutively incubated 60 min with anti-γH2AX antibody (1:500, clone JBW301, Merck Millipore) and Alexa Fluor 594 goat anti-mouse IgG1 (1:400, Molecular Probes^®^/Life Technologies, Darmstadt, Germany) for 30 min. The slides were mounted with Vectashield^®^ containing anti-4′,6-diamidino-2-phenylindole (DAPI; Vector Laboratories, Inc., Burlingame, CA, USA). The foci were visualised with an Eclipse TE300 inverted microscope (Nikon, Tokyo, Japan). At a magnification of 1000×, the foci of 50 cells per chamber were counted; two chambers per IR dose.

### 4.7. Statistical Analysis

All data was presented as the mean ± standard deviation (SD) or standard error of mean (SEM). The normality of the distribution of each parameter was assessed using the Anderson–Darling test. Where normality assumptions were not met, data were logarithmically transformed. To identify differences between data sets, the two-tailed Student’s *t*-test was performed. To compare a variable under different conditions, the two-way ANOVA was performed, followed by a Bonferroni post-hoc test performed with SigmaPlot (Version 13.0, Systat Software GmbH). Significance was assessed at *p* < 0.05 (*: *p* < 0.05, **: *p* < 0.01; ***: *p* < 0.001). For comparative analysis of one dataset to a fixed value, the one-sample *t*-test was used. Here, a *p* value of <0.02 was considered a significant difference (*: *p* < 0.02, **: *p* < 0.01; ***: *p* < 0.002). 

## Abbreviations

AT	adipose tissue
ADSCs	adipose-derived stem cells
BMSCs	bone marrow-derived stem cells
CDK	cyclin-dependent kinase
DSBs	double-strand breaks
ESCs	embryonic stem cells
iPSCs	induced pluripotent stem cells
IR	irradiation
MMP-2	matrix metalloprotenase-2
pADSCs	pooled adipose-derived stem cells
PE	plating efficiency
γH2AX	phosphorylated H2AX
SD	standard deviation
SF	survival fraction
VEGF	vascular endothelial growth factor

## Appendix A

**Table A1:** Comparison of pADSC survival fractions for each dose with those of MCF-7, MCF10A, and ZR-75-1 cells.

	Irradiation Dose (Gy)	Survival Fraction ± Standard Deviation	*p* Value ^1^
pADSCs	0.5	0.88 ± 0.09	
	1	0.79 ± 0.07	
	2	0.64 ± 0.05	
	4	0.26 ± 0.08	
	6	0.05 ± 0.01	
MCF-7 cells	**2**	0.52 ± 0.06	**0.01811 ***
	4	0.21 ± 0.05	0.29717
	6	0.06 ± 0.02	0.51909
MCF10A cells	**2**	**0.85** ± 0.05	**0.00722 ****
	**4**	**0.61** ± 0.04	**0.00165 ****
	**6**	**0.23** ± 0.01	**0.00244 ****
ZR-75-1 cells	2	0.48 ± 0.19	0.19339
	**4**	**0.10** ± 0.05	**0.01072 ***
	**6**	**0.01** ± 0.01	**0.00069 *****

^1^ Student’s *t*-test was used to compare survival fractions of pADSCs for each dose with survival fractions of MCF-7, MCF10A, and ZR-75-1 cells; bolded datasets represent significant differences to the dataset of pADSCs, asterisks illustrate significance: * *p* < 0.05; ** *p* < 0.01; *** *p* < 0.001.

**Table A2:** Population doubling times (PDT) of ADSCs within an incubation time of 48 to 240 h with special emphasis on the PDT of pooled cells and pooled data (bolded).

	PDT (48–240 h) ± Standard Deviation
**pooled cells**	**96.5 ± 8.0 h**
donor 1	110.7 ± 33.1 h
donor 2	73.4 ± 7.3 h
donor 3	79.2 ± 19.0 h
donor 4	82.2 ± 20.4 h
donor 5	104.1 ± 27.2 h
donor 6	107.9 ± 23.3 h
donor 7	75.6 ± 9.2 h
donor 8	97.3 ± 18.9 h
donor 9	113.0 ± 12.2 h
donor 10	179.6 ± 75.8 h
**pooled data**	**102.3 ± 29.5 h**
